# Endoscopic ultrasound-guided biliary drainage: a review

**DOI:** 10.1007/s12328-014-0467-5

**Published:** 2014-03-06

**Authors:** Takuji Iwashita, Shinpei Doi, Ichiro Yasuda

**Affiliations:** First Department of Internal Medicine, Gifu University Hospital, 1-1 Yanagido, Gifu, 501-1194 Japan

**Keywords:** Endoscopic ultrasound, Endoscopic retrograde cholangiopancreatography, Biliary drainage, Rendezvous, Antegrade

## Abstract

Endoscopic retrograde cholangiography (ERCP) is widely used as a first-line therapy for biliary drainage. ERCP occasionally fails owing to anatomical or technical problems, despite high reported success rates. Endoscopic ultrasound-guided biliary drainage (EUS-BD) has recently emerged as an effective alternative biliary drainage method after unsuccessful ERCP. EUS-BD can be essentially divided into 3 different techniques—(1) EUS-guided transluminal biliary drainage including choledocoduodenostomy and hepaticogastrostomy, (2) EUS-rendezvous technique, and (3) EUS-antegrade approach. Here, we focus on the current status of EUS-BD in light of these 3 different techniques.

## Introduction

Endoscopic retrograde cholangiopancreatography (ERCP) is widely used in current medical practice as a biliary drainage method for biliary obstruction. ERCP occasionally fails owing to anatomical or technical problems such as upper intestinal obstruction, surgically altered anatomy, periampullary diverticulum, or periampullary tumor infiltration, despite a >90 % success rate in most reports. Percutaneous transhepatic biliary drainage (PTBD) or surgical interventions are conventionally performed as alternative biliary drainage methods after unsuccessful ERCP. However, both PTBD and surgical interventions are associated with considerable morbidity and mortality [1–3].

Endoscopic ultrasound-guided biliary drainage (EUS-BD) was first reported in 2001 by Giovannini et al. [4]. Following this report, many groups reported the efficacy of EUS-BD as an alternative biliary drainage method after unsuccessful ERCP. Reported EUS-BD procedures are divided into 3 techniques—(1) EUS-guided transluminal biliary drainage including choledocoduodenostomy (EUS-CDS) and hepaticogastrostomy (EUS-HGS), (2) EUS-rendezvous technique (EUS-RV), and (3) EUS-antegrade approach (EUS-AG). Here, we focus on the status of EUS-BD in light of these 3 different techniques.

## EUS-transluminal biliary drainage

### Summary of the procedure

In EUS-transluminal biliary drainage, the biliary duct is accessed under EUS guidance followed by guidewire placement and fistula dilation. A stent is then deployed between the biliary duct and intestine to create a permanent fistula for biliary drainage. This procedure can be performed in patients with either endoscopically accessible or inaccessible papilla; however, its indication should be limited in cases of unresectable malignant biliary obstruction, given the feature of permanent fistula creation.

### Actual technique

**Fig. 1:**
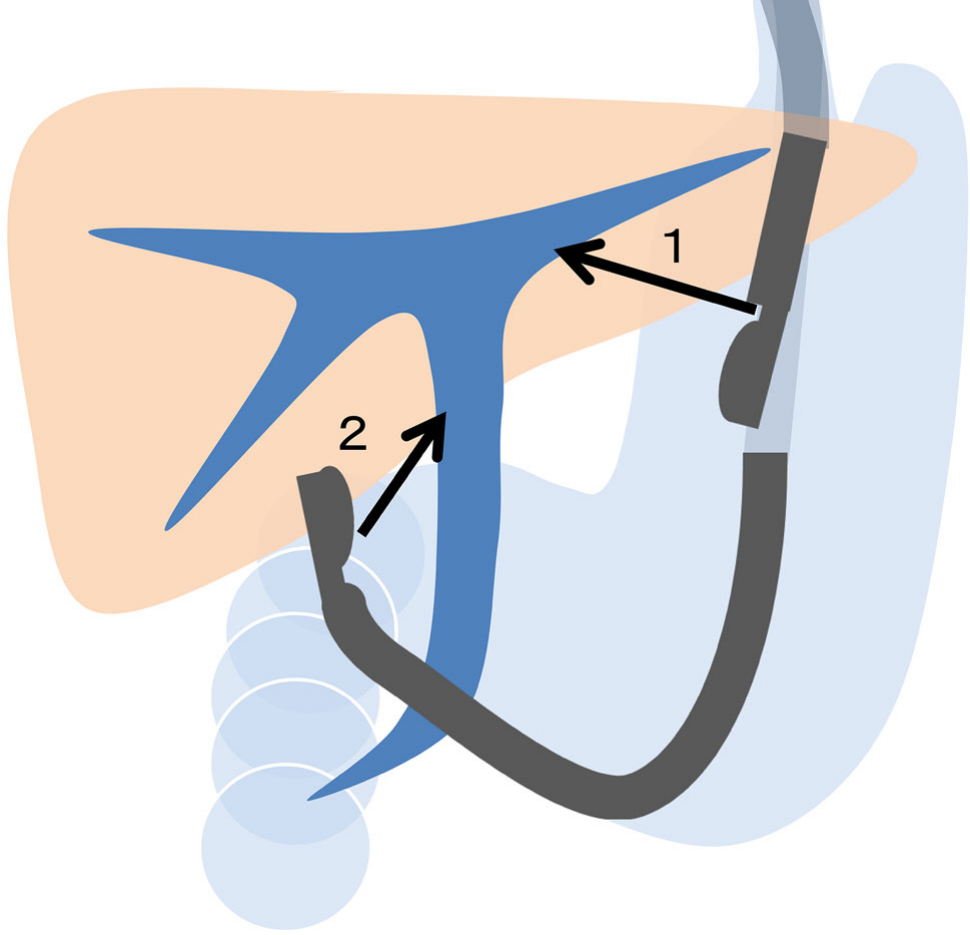
Access routes of endoscopic ultrasound-guided transluminal biliary drainage. *1* Hepaticogastrostomy; *2* choledocoduodenostomy

For EUS-transluminal biliary drainage, the biliary duct is punctured from the upper intestine under EUS guidance followed by cholangiography to ensure proper puncture of the biliary duct and delineate its configuration. A guidewire is then placed into the biliary system and dilation of the needle tract is performed. In this step, a fine needle aspiration (FNA) needle, needle knife, or fistulotome can be used for bile duct puncture with fistula creation performed using a bougie dilator, needle knife, balloon, stent retriever, or diathermic sheath over the guidewire. Finally, a stent is deployed at the fistula between the biliary duct and the intestine for biliary drainage. In terms of access route, this technique is divided further into HGS, in which the fistula is made between the stomach and intrahepatic bile duct (IHBD) of the left lobe, and CDS, in which the fistula is created between the duodenal bulb (D1) and extrahepatic bile duct (EHBD) (Fig. 1).

### Literature review and assessment

Since the first report of EUS-CDS in 2001 by Giovannini et al. [4], multiple groups reported the efficacy of EUS-CDS and HGS as alternative drainage methods to PTBD or surgery after unsuccessful ERCP. Published data for EUS-CDS and HGS show overall technical success rates of 94 and 87 % with overall early complication rates of 19 and 27 %, respectively [4–40] (Tables 1, 2). The published overall technical success rates are similarly high for both techniques, although various biliary access and fistula dilation methods are used depending on preference of each institution and endoscopist. Regarding stents, there is a tendency to use a covered self-expandable metallic stent (CMS), instead of a plastic stent (PS), especially in later studies. Performing CMS placement to maintain the fistula for bile drainage is sensible, as a CMS can potentially prolong the stent patency period compared with a PS. Furthermore, radial expansion of a CMS can, hypothetically, minimize the possibility for complications such as bile peritonitis or pneumoperitoneum because the fistula is immediately sealed by the CMS itself. However, stent migration is a serious complication that can still occur even with the use of a CMS, especially soon after the procedure. Development of specially designed stents for these procedures and further assessment of the methodology of biliary access and fistula dilation are mandatory for generalization of EUS-CDS and HGS.

**Table 1:** Published data on EUS-guided choledocoduodenostomy

References	Access route (*n*)	Access method	Dilation method	Stent	Technical success rate, % (*n*)	Early complication rate, % (*n*)	Complications, *n*
Giovannini et al. [4]	CDS (1)	NK	BD	PS	100 (1/1)	0 (0/1)	None
Burmester et al. [5]	CDS (2)	Fistulotome	None	PS	50 (1/2)	50 (1/2)	Bile peritonitis 1
Puspok et al. [6]	CDS (5)	NK	None	PS	80 (4/5)	0 (0/5)	None
Yamao et al. [7]	CDS (2)	NK	BD	PS	100 (2/2)	0 (0/2)	None
Fujita et al. [8]	CDS (1)	19G	BD	PS	100 (1/1)	0 (0/1)	None
Ang et al. [9]	CDS (2)	19G	NK, BD	PS	100 (2/2)	50 (1/2)	Pneumoperitoneum 1
Tarantino et al. [10]	CDS (4)	19G, 22G	NK, balloon	PS	100 (4/4)	0 (0/4)	None
Yamao et al. [11]	CDS (5)	NK	BD	PS	100 (5/5)	20 (1/5)	Pneumoperitoneum 1
Itoi et al. [12]	CDS (4)	19G, NK	BD	PS, NBD	100 (4/4)	25 (1/4)	Bile peritonitis 1
Brauer et al. [13]	CDS (3)	19G, 22G	NK	PS	100 (3/3)	33 (1/3)	Pneumoperitoneum 1
Horaguchi et al. [14]	CDS (8)	19G	BD, balloon	PS, NBD	100 (8/8)	13 (1/8)	Peritonitis 1
Hanada et al. [15]	CDS (4)	19G	BD	PS	100 (4/4)	0 (0/4)	None
Park et al. [16]	CDS (4), CGS (1)	19G	NK, BD	CMS	100 (5/5)	0 (0/5)	None
Iwamuro et al. [17]	CDS (5)	NK	BD	PS	100 (5/5)	20 (1/5)	Severe abdominal pain and fever 1
Siddiqui et al. [18]	CDS (8)	19G	NK	CMS	100 (8/8)	25 (2/8)	Duodenal perforation 1, abdominal pain 1
Belletrutti et al. [19]	CDS (4)	19G	Balloon	PS, CMS	100 (4/4)	0 (0/4)	None
Hara et al. [20]	CDS (18)	NK	BD	PS	94 (17/18)	17 (3/18)	Peritonitis 2, hemobilia 1
Komaki et al. [21]	CDS (15)	19G, NK	BD	PS	93 (14/15)	47 (7/15)	Cholangitis 4, peritonitis 2, stent migration 1
Ramirez-Luna et al. [22]	CDS (9)	19G	NK, BD, balloon	PS	89 (8/9)	11 (1/9)	Biloma
Park et al. [23]	CDS (24)	19G	NK, BD	PS, CMS	92 (24/26)	19 (5/26)	n/a
Fabbri et al. [24]	CDS (15)	19G	NK, balloon	CMS	80 (12/15)	7 (1/15)	Pneumoperitoneum 1
Kawakubo et al. [25]	CDS (1)	19G	BD, balloon	PS	100 (2/2)	0 (1/1)	None
Katanuma et al. [26]	CDS (1)	19G	NK, BD	PS	100 (1/1)	0 (0/1)	None
Attasaranya et al. [27]	CDS (9)	19G	BD	PS, CMS	56 (5/9)	44 (4/9)	n/a
Artifon et al. [28]	CDS (13)	19G	NK, BD	CMS	100 (13/13)	15 (2/13)	Bleeding 1, bile leak 1
Kim et al. [29]	CDS (9)	19G	NK, BD	CMS	100 (9/9)	50 (5/10)	Pneumoperitoneum 2, migration 2, peritonitis 1
Song et al. [30]	CDS (15)	19G	NK, BD	CMS	87 (13/15)	23 (3/15)	Pneumoperitoneum 2, cholangitis 1
Vila et al. [31]	CDS (26)	n/a	n/a	n/a	86 (19/26)	15 (4/26)	Biloma 1, bleeding 1, pancreatitis 1, cholangitis 1
Tonozuka et al. [32]	CDS (4) CGS (1)	19G	BD, balloon, DS	CMS	100 (5/5)	0 (0/5)	None
Khashab et al. [33]	CDS (15)	19G, 22G	BD, balloon	PS, CMS	100 (20/20)	n/a (n/a)	n/a
Kawakubo et al. [34]	CDS (44)	19G, NK	BD, balloon, SR, DS	PS, CMS	95 (42/44)	14 (6/44)	Bile leak 3, stent misplacement 1, bleeding 1, pneumoperitoneum 1, perforation 1
Hara et al. [35]	CDS (18)	NK	BD	CMS	94 (17/18)	11 (2/18)	Peritonitis 2
Overall					94 (282/300)	19 (53/280)	

*CDS* choledocoduodenostomy, *CGS* choledocogastrostomy, *19G* 19-gauge FNA needle, *22G* 22-gauge FNA needle, *NK* needle knife, *CN* coagulation needle, *SR* stent retriever, *DS* diathermic sheath, *PS* plastic stent, *CMS* covered self-expandable metallic stent

**Table 2:** Published data on EUS-guided hepaticogastrostomy

References	Access route (*n*)	Access method	Dilation method	Stent	Technical success rate, % (*n*)	Early complication rate, % (*n*)	Complications, *n*
Burmester et al. [5]	HGS (1), HJS (1)	Fistulotome	None	PS	100 (2/2)	0 (0/2)	None
Giovannini et al. [36]	HGS (1)	19G	NK	PS	100 (1/1)	0 (0/1)	None
Artifon et al. [37]	HGS (1)	19G	BD, balloon	CMS	100 (1/1)	0 (0/1)	None
Will et al. [38]	HES (1), HGS (4), HJS (3)	19G	BD, balloon	PS, CMS	88 (7/8)	25 (2/8)	Cholangitis and pain 1, pain 1
Bories et al. [39]	HGS (11)	19G, 22G	Cystotome	PS, CMS	91 (10/11)	36 (4/11)	Early stent occlusion 1, transient ileus 1, biloma 1, cholangitis 1
Park et al. [16]	HGS (8), HES (1)	19G	BD, NK	CMS	100 (9/9)	22 (2/9)	Pneumoperitoneum 2
Iwamuro et al. [17]	HGS (2)	NK	BD	PS	100 (2/2)	50 (1/2)	Bile leak and pneumoperitoneum 1
Park et al. [40]	HGS (5)	NK	BD	CMS	100 (5/5)	0 (0/5)	None
Belletrutti et al. [19]	HGS (3)	19G	Balloon	PS, CMS	67 (2/3)	0 (0/3)	None
Ramirez-Luna et al. [22]	HGS (2)	19G	NK, BD	PS	100 (2/2)	50 (1/2)	Stent migration 1
Park et al. [23]	HGS (31)	19G	NK, BD	PS, CMS	100 (31/31)	19 (6/31)	n/a
Fabbri et al. [24]	HGS (1)	19G	NK, balloon	CMS	0 (0/1)	0 (0/1)	None
Attasaranya et al. [27]	HGS (16)	19G	BD	PS, CMS	81 (13/16)	38 (6/16)	n/a
Kim et al. [29]	HGS (4)	19G	NK, BD	CMS	75 (3/4)	50 (2/4)	Abdominal pain 1, stent migration 1
Vila et al. [31]	HGS (34)	n/a	n/a	n/a	65 (22/34)	29 (11/34)	Biloma 3, bleeding 3, perforation 2, liver hematoma 1, abscess 1
Tonozuka et al. [32]	HGS (3)	19G	BD, balloon, CN	CMS	100 (3/3)	0 (0/3)	None
Khashab et al. [33]	HGS (3), HES (2)	19G, 22G	BD, balloon	PS, CMS	100 (5/5)	n/a (n/a)	n/a
Kawakubo et al. [34]	HGS (20)	19G	BD, balloon, NK	PS, CMS	95 (19/20)	30 (6/20)	Bile leak 2, stent misplacement 2, bleeding 1, cholangitis 1, biloma 1
Overall					87 (137/158)	27 (41/153)	

*HGS* hepaticogastrostomy, *HJS* hepaticojejunostomy, *HES* hepaticoesophagostomy, *19G* 19-gauge FNA needle, *22G* 22-gauge FNA needle, *NK* needle knife, *CN* coagulation needle, *PS* plastic stent, *CMS* covered self-expandable metallic stent

## EUS-rendezvous technique

### Summary of the procedure

In EUS-RV, the biliary duct is accessed under EUS and fluoroscopic guidance with the creation of a temporary fistula followed by guidewire placement via the biliary duct and ampulla into the duodenum. After guidewire placement, ERCP is re-attempted using the EUS-placed guidewire. The guidewire is removed once biliary cannulation is obtained. Therefore, EUS-RV should be attempted for patients with an endoscopically accessible ampulla after failed biliary cannulation in conventional ERCP.

### Actual technique

After failed biliary cannulation in ERCP, a duodenoscope is exchanged for a linear EUS scope. The biliary system is visualized from the stomach or duodenum with color Doppler to detect any vessels interposing on the puncture route. The bile duct is then punctured using an FNA needle, in which the stylet is removed and the contrast is primed, followed by guidewire placement into the biliary system through the needle after confirmation of proper puncture of bile duct with cholangiogram. Either a 19- or 22-gauge (G) FNA needle can be used for EUS-RV. A 19-G needle allows a guidewire of up to 0.035 inches to pass through the needle, whereas a 22-G needle only allows a 0.018-inch guidewire to pass through. The guidewire is then manipulated into the duodenum via the obstruction and the ampulla. After the needle is withdrawn inside the outer sheath, the EUS scope and needle are removed while maintaining the guidewire in place. The duodenoscope is reinserted along with the EUS-placed guidewire to the ampulla, where the EUS-placed guidewire exits from the biliary orifice. Biliary cannulation is reattempted along with the guidewire. As another means of obtaining biliary cannulation, the distal end of the guidewire is grasped with forceps or a snare and the guidewire is pulled out through the mouth with the scope or through its accessory channel. An ERCP cannula inserted over the guidewire or the duodenoscope is back-loaded over the guidewire and re-advanced to the ampulla if the guidewire is pulled out through the mouth. Finally, endoscopic biliary stenting is accomplished as planned.

### Literature review and assessment

Published EUS-RV data are shown in Table 3 [10, 33, 41–51]. The overall success rate of EUS-RV is 81 % with a complication rate of 10 %. One of the most challenging aspects of EUS-RV is guidewire manipulation, in which the guidewire has to pass through the long rigid needle, biliary ducts, obstruction, and ampulla [52]. EUS-RV can be divided into IHBD and EHBD approaches. The EHBD approach can be performed with 2 scope positions, the push (long) and pull (short) positions, in terms of the scope shapes during EUS-RV (Table 4). In the IHBD approach, the biliary duct can be accessed from the stomach using the straight scope position, which eases needle maneuverability, although correct biliary puncture may be difficult in patients with insufficient IHBD dilation. Furthermore, the longer distance between the access point and the ampulla decreases the pushability and torque transmission of the guidewire needed to pass though the downstream resistance. In the EHBD approach using the push scope position, the distance between the access point and the ampulla is short because the EHBD is punctured from D1; however, the loop of the scope inside the stomach may impair maneuverability of the needle, and access to the EHBD with the needle directed toward the hepatic hilar makes guidewire manipulation toward the ampulla side difficult. On the other hand, in the EHBD approach using the pull scope position, biliary access is made with a very short distance and needle direction toward the ampulla. However, this approach might be difficult in patients with distal bile duct obstruction, such as those with pancreatic head cancer, because the scope position might be lost from D2 when the scope is pulled to puncture the EHBD above the obstruction. Some groups prefer the IHBD approach, as it is considered to have a lower risk of bile leakage than the EHBD approach [44, 47]. Theoretically, the IHBD approach may reduce the risk of bile leakage because the liver parenchyma around the bile duct can tamponade the temporal fistula. However, we believe that selection of approach routes that maximize the success rate is the most important factor to reduce the complications associated with bile leakage, as proper biliary drainage can reduce bile leakage and treat bile peritonitis. Careful selection of the biliary duct access point and scope position for feasible guidewire manipulation with consideration of the aforementioned factors is important to assure successful EUS-RV.

**Table 3:** Published data on EUS-guided rendezvous technique

References	EHBD approach	IHBD approach	Overall		Complications, *n*
	Success rate, % (n)	Success rate, % (n)	Success rate, % (n)	Complication rate, % (n)	
Tarantino et al. [10]	50 (4/8)	–	50 (4/8)	13 (1/8)	Death due to LC 1
Maranki et al. [42, 44, 45]^c^	57 (8/14)^a^	65 (26/40)^a^	63 (34/49)^a^	16 (8/49)	Abdominal pain 1, pneumoperitoneum 4, bleeding 1, biliary peritonitis 1, aspiration pneumonia 1
Kim et al. [41, 43, 46]^c^	80 (12/15)	–	80 (12/15)	13 (2/15)	Sepsis 1, pancreatitis 1
Shah et al. [47]	n/a (n/a)	n/a (n/a)	74 (37/50)	8 (4/50)	Pancreatitis 2, bile leak 1, perforation 1
Iwashita et al. [48]	81 (25/31)	44 (4/9)	73 (29/40)	13 (5/40)	Abdominal pain 1, pancreatitis 2, pneumoperitoneum 1, sepsis/death 1^b^
Dhir et al. [49]	98 (57/58)	–	98 (57/58)	3 (2/58)	Extravasation of contrast 2
Kawakubo et al. [50]	100 (9/9)	100 (5/5)	100 (14/14)	14 (2/14)	Pancreatitis 1, bile peritonitis 1
Park et al. [51]	93 (13/14)	50 (3/6)	80 (16/20)	10 (2/20)	Pancreatitis 1, bile peritonitis 1
Khashab et al. [33]	100 (11/11)	100 (2/2)	100 (13/13)	15 (2/13)	Pancreatitis 1, cholecystitis 1
Overall	87 (139/160)	65 (40/62)	81 (215/267)	11 (24/217)	

*EHBD* extra hepatic bile duct, *IHBD* intra-hepatic bile duct, *LC* liver cirrhosis

^a^Including 5 patients converted from IHBD approach

^b^Assessed unrelated to the procedure

^c^Overlapping references

**Table 4:** Comparison of approach routes during EUS-rendezvous technique

	IHBD	EHBD	
Scope position	Straight	Push (long)	Pull (short)
Schema			
Puncture site	Stomach	D1	D2
Scope stability	Stable	Stable	Unstable
Needle maneuverability	Easy	Difficult	Normal
Diameter of bile duct	Small	Large	Large
Needle direction	Ampulla	Hepatic hilar	Ampulla
Distance to papilla	Long	Short	Very short

*IHBD* intra hepatic bile duct, *EHBD* extra hepatic bile duct, *D1* duodenal bulbs, *D2* 2nd portion of the duodenum

## EUS-guided antegrade treatments

### Summary of the procedure

In EUS-AG, the IHBD is accessed from the upper intestine with creation of a temporary fistula between the intestine and IHBD. After dilation of the fistula, stent placement or balloon dilation are performed for biliary obstruction through the fistula without the endoscope reaching the ampulla. This technique is suitable for biliary obstruction in patients with surgically altered anatomy or upper intestinal obstruction, in which reaching the biliary orifice endoscopically is impossible or cumbersome.

### Actual technique

After careful examination of the left lobe of the liver using color Doppler to detect any interposing vessels, the IHBD is punctured from the intestine using an FNA needle primed with contrast agent. Correct biliary puncture is confirmed with bile aspiration and cholangiography through the needle. A guidewire is then inserted into the biliary system through the needle followed by removal of the needle and dilation of the fistula using a bougie dilator over the guidewire. The guidewire is then manipulated into the intestine through the ampulla or anastomosis, with coordinated movements of the guidewire and dilator inside the biliary system. A self-expandable metallic stent is deployed to the malignant biliary obstruction or balloon dilation is performed for a benign biliary stricture in an antegrade fashion. Finally, all devices are removed after confirmation that bile flows well through the stent or stricture.

### Literature review and assessment

Only several reports exist regarding one-step EUS-AG. The overall success and complication rates of EUS-AG are 77 and 5 %, respectively [47, 51, 53–56] (Table 5). EUS-AG also requires complicated guidewire manipulation, similar to EUS-RV in which the guidewire has to be placed through the FNA needle, via the biliary duct, obstruction, and ampulla, into the duodenum. However, in EUS-AG, the guidewire can be manipulated with coordinated movement of the catheter inside the biliary duct, like PTBD, once the fistula is dilated with a bougie dilator. On the other hand, a major concern in EUS-AG is the possibility of bile leakage into the peritoneal cavity through the temporally dilated fistula after the procedure. In the reviewed literatures, however, no cases of biliary peritonitis have been reported, although further study is needed to evaluate possible complications.

**Table 5:** Published data on EUS-guided antegrade treatments for biliary drainage

References	Success rate, % (n)	Complication rate, % (n)	Complications, *n*
Nguyen-Tang et al. [53]	100 (5/5)	0 (0/5)	None
Artifon et al. [54]	100 (1/1)	0 (0/1)	None
Park et al. [55]	100 (1/1)	0 (0/1)	None
Shah et al. [47]	81 (13/16)	6 (1/16)	Hepatic hematoma 1
Iwashita et al. [56]	100 (2/2)	50 (1/2)	Pancreatitis 1
Park et al. [51]	57 (8/14)	0 (0/14)	None
Overall	77 (30/39)	5 (2/39)	

## Selection of techniques in EUS-guided biliary drainage

All 3 EUS-BD techniques require an experienced endoscopist in both the EUS and ERCP procedures as well as the capability of EUS and fluoroscopy. Availability of alternative biliary decompression, such as PTBD or surgery, is also critical to minimize the risk of bile leakage into the peritoneal cavity in preparation for unsuccessful EUS-BD. The indication for EUS-BD should be decided carefully by taking into account the condition and needs of the patient as well as the endoscopist and facilities available.

Once the decision to perform EUS-BD is made, selection of situation-specific EUS-BD techniques should be made, although each EUS-BD technique has overlapping indications and there is no guideline for selection of techniques. Khashab et al. [33] compared outcomes of EUS-RV and EUS-transluminal biliary drainage using a standardized approach, in which EUS-RV is initially attempted if the ampulla is accessible, followed by EUS-transluminal biliary drainage as a salvage procedure for cases of unsuccessful guidewire placement into the intestine; EUS-transluminal biliary drainage is the first option if the ampulla is inaccessible. Their results suggested that both techniques seem to be equally effective and safe with their standardized approach and EUS-transluminal biliary drainage is a reasonable alternative to EUS-RV. Park et al. [51] also conducted a study using their treatment procedure with an enhanced guidewire manipulation protocol including EUS-RV for patients with an accessible ampulla or EUS-AG for patients with surgically altered anatomy. EUS-AG was considered a first-line intervention for patients with an inaccessible ampulla after failed ERCP, with the exception of patients with duodenal invasion. For patients with duodenal invasion, EUS-transluminal biliary drainage was performed after duodenal stent placement, as ampullary access and transampullary drainage are impossible. In this study, a favorable success rate and acceptable adverse event rate were achieved with their enhanced procedure. As these two studies indicated, it might be important to apply different EUS-BD techniques depending on patient condition and progress of the procedure.

**Fig. 2:**
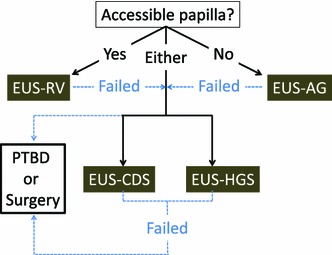
Proposed treatment procedure using endoscopic ultrasound-guided biliary drainage after unsuccessful endoscopic retrograde cholangiography

Given the results of previous studies regarding EUS-BD, our proposed treatment algorithm using EUS-BD after unsuccessful ERCP is shown in Fig. 2. EUS-RV can be a first-line EUS-BD technique in patients with an endoscopically accessible ampulla. If endoscopic access to the ampulla is impossible or difficult (e.g., surgically altered anatomy), EUS-AG is a suitable option. EUS-CDS and HGS can be used for either an accessible or inaccessible ampulla, but have a good indication for an ampulla with tumor invasion. If all EUS-guided managements fail, PTBD or surgery should be considered the next step to minimize potential complications.

## Conclusion

EUS-BD is a feasible salvage technique for unsuccessful ERCP, although further studies are needed to compare the efficacy and safety between EUS-BD and PTBD and to examine a treatment procedure using EUS-BD techniques.

## References

[1] Smith AC, Dowsett JF, Russell RC, Hatfield AR, Cotton PB (1994). Randomised trial of endoscopic stenting versus surgical bypass in malignant low bileduct obstruction. Lancet.

[2] Kama NA, Coskun T, Yuksek YN, Yazgan A (1999). Factors affecting post-operative mortality in malignant biliary tract obstruction. Hepatogastroenterology.

[3] Beissert M, Wittenberg G, Sandstede J, Beer M, Tschammler A, Burghardt W (2002). Metallic stents and plastic endoprostheses in percutaneous treatment of biliary obstruction. Z Gastroenterol.

[4] Giovannini M, Moutardier V, Pesenti C, Bories E, Lelong B, Delpero JR (2001). Endoscopic ultrasound-guided bilioduodenal anastomosis: a new technique for biliary drainage. Endoscopy.

[5] Burmester E, Niehaus J, Leineweber T, Huetteroth T (2003). EUS-cholangio-drainage of the bile duct: report of 4 cases. Gastrointest Endosc.

[6] Puspok A, Lomoschitz F, Dejaco C, Hejna M, Sautner T, Gangl A (2005). Endoscopic ultrasound guided therapy of benign and malignant biliary obstruction: a case series. Am J Gastroenterol.

[7] Yamao K, Sawaki A, Takahashi K, Imaoka H, Ashida R, Mizuno N (2006). EUS-guided choledochoduodenostomy for palliative biliary drainage in case of papillary obstruction: report of 2 cases. Gastrointest Endosc.

[8] Fujita N, Noda Y, Kobayashi G, Ito K, Obana T, Horaguchi J (2007). Histological changes at an endosonography-guided biliary drainage site: a case report. World J Gastroenterol.

[9] Ang TL, Teo EK, Fock KM (2007). EUS-guided transduodenal biliary drainage in unresectable pancreatic cancer with obstructive jaundice. JOP.

[10] Tarantino I, Barresi L, Repici A, Traina M (2008). EUS-guided biliary drainage: a case series. Endoscopy.

[11] Yamao K, Bhatia V, Mizuno N, Sawaki A, Ishikawa H, Tajika M (2008). EUS-guided choledochoduodenostomy for palliative biliary drainage in patients with malignant biliary obstruction: results of long-term follow-up. Endoscopy.

[12] Itoi T, Itokawa F, Sofuni A, Kurihara T, Tsuchiya T, Ishii K (2008). Endoscopic ultrasound-guided choledochoduodenostomy in patients with failed endoscopic retrograde cholangiopancreatography. World J Gastroenterol.

[13] Brauer BC, Chen YK, Fukami N, Shah RJ (2009). Single-operator EUS-guided cholangiopancreatography for difficult pancreaticobiliary access (with video). Gastrointest Endosc.

[14] Horaguchi J, Fujita N, Noda Y, Kobayashi G, Ito K, Obana T (2009). Endosonography-guided biliary drainage in cases with difficult transpapillary endoscopic biliary drainage. Dig Endosc.

[15] Hanada K, Iiboshi T, Ishii Y (2009). Endoscopic ultrasound-guided choledochoduodenostomy for palliative biliary drainage in cases with inoperable pancreas head carcinoma. Dig Endosc.

[16] Park do H, Koo JE, Oh J, Lee YH, Moon SH, Lee SS (2009). EUS-guided biliary drainage with one-step placement of a fully covered metal stent for malignant biliary obstruction: a prospective feasibility study. Am J Gastroenterol.

[17] Iwamuro M, Kawamoto H, Harada R, Kato H, Hirao K, Mizuno O (2010). Combined duodenal stent placement and endoscopic ultrasonography-guided biliary drainage for malignant duodenal obstruction with biliary stricture. Dig Endosc.

[18] Siddiqui AA, Sreenarasimhaiah J, Lara LF, Harford W, Lee C, Eloubeidi MA (2011). Endoscopic ultrasound-guided transduodenal placement of a fully covered metal stent for palliative biliary drainage in patients with malignant biliary obstruction. Surg Endosc.

[19] Belletrutti PJ, DiMaio CJ, Gerdes H, Schattner MA (2011). Endoscopic ultrasound guided biliary drainage in patients with unapproachable ampullae due to malignant duodenal obstruction. J Gastrointest Cancer.

[20] Hara K, Yamao K, Niwa Y, Sawaki A, Mizuno N, Hijioka S (2011). Prospective clinical study of EUS-guided choledochoduodenostomy for malignant lower biliary tract obstruction. Am J Gastroenterol.

[21] Komaki T, Kitano M, Sakamoto H, Kudo M (2011). Endoscopic ultrasonography-guided biliary drainage: evaluation of a choledochoduodenostomy technique. Pancreatology.

[22] Ramirez-Luna MA, Tellez-Avila FI, Giovannini M, Valdovinos-Andraca F, Guerrero-Hernandez I, Herrera-Esquivel J (2011). Endoscopic ultrasound-guided biliodigestive drainage is a good alternative in patients with unresectable cancer. Endoscopy.

[23] Park do H, Jang JW, Lee SS, Seo DW, Lee SK, Kim MH (2011). EUS-guided biliary drainage with transluminal stenting after failed ERCP: predictors of adverse events and long-term results. Gastrointest Endosc.

[24] Fabbri C, Luigiano C, Fuccio L, Polifemo AM, Ferrara F, Ghersi S (2011). EUS-guided biliary drainage with placement of a new partially covered biliary stent for palliation of malignant biliary obstruction: a case series. Endoscopy.

[25] Kawakubo K, Isayama H, Nakai Y, Sasahira N, Kogure H, Sasaki T (2012). Simultaneous duodenal metal stent placement and EUS-guided choledochoduodenostomy for unresectable pancreatic cancer. Gut Liver.

[26] Katanuma A, Maguchi H, Osanai M, Takahashi K (2012). Endoscopic ultrasound-guided biliary drainage performed for refractory bile duct stenosis due to chronic pancreatitis: a case report. Dig Endosc.

[27] Attasaranya S, Netinasunton N, Jongboonyanuparp T, Sottisuporn J, Witeerungrot T, Pirathvisuth T (2012). The spectrum of endoscopic ultrasound intervention in biliary diseases: a single center’s experience in 31 cases. Gastroenterol Res Pract.

[28] Artifon EL, Aparicio D, Paione JB, Lo SK, Bordini A, Rabello C (2012). Biliary drainage in patients with unresectable, malignant obstruction where ERCP fails: endoscopic ultrasonography-guided choledochoduodenostomy versus percutaneous drainage. J Clin Gastroenterol.

[29] Kim TH, Kim SH, Oh HJ, Sohn YW, Lee SO (2012). Endoscopic ultrasound-guided biliary drainage with placement of a fully covered metal stent for malignant biliary obstruction. World J Gastroenterol.

[30] Song TJ, Hyun YS, Lee SS, Park do H, Seo DW, Lee SK (2012). Endoscopic ultrasound-guided choledochoduodenostomies with fully covered self-expandable metallic stents. World J Gastroenterol.

[31] Vila JJ, Perez-Miranda M, Vazquez-Sequeiros E, Abadia MA, Perez-Millan A, Gonzalez-Huix F (2012). Initial experience with EUS-guided cholangiopancreatography for biliary and pancreatic duct drainage: a Spanish national survey. Gastrointest Endosc.

[32] Tonozuka R, Itoi T, Sofuni A, Itokawa F, Moriyasu F (2013). Endoscopic double stenting for the treatment of malignant biliary and duodenal obstruction due to pancreatic cancer. Dig Endosc.

[33] Khashab MA, Valeshabad AK, Modayil R, Widmer J, Saxena P, Idrees M (2013). EUS-guided biliary drainage by using a standardized approach for malignant biliary obstruction: rendezvous versus direct transluminal techniques (with videos). Gastrointest Endosc.

[34] Kawakubo K, Isayama H, Kato H, Itoi T, Kawakami H, Hanada K, et al. Multicenter retrospective study of endoscopic ultrasound-guided biliary drainage for malignant biliary obstruction in Japan. J Hepatobiliary Pancreat Sci. 2013 (Epub 2013/09/13). 10.1002/jhbp.27 24026963

[35] Hara K, Yamao K, Hijioka S, Mizuno N, Imaoka H, Tajika M (2013). Prospective clinical study of endoscopic ultrasound-guided choledochoduodenostomy with direct metallic stent placement using a forward-viewing echoendoscope. Endoscopy.

[36] Giovannini M, Dotti M, Bories E, Moutardier V, Pesenti C, Danisi C (2003). Hepaticogastrostomy by echo-endoscopy as a palliative treatment in a patient with metastatic biliary obstruction. Endoscopy.

[37] Artifon EL, Chaves DM, Ishioka S, Souza TF, Matuguma SE, Sakai P (2007). Echoguided hepatico-gastrostomy: a case report. Clinics (Sao Paulo).

[38] Will U, Thieme A, Fueldner F, Gerlach R, Wanzar I, Meyer F (2007). Treatment of biliary obstruction in selected patients by endoscopic ultrasonography (EUS)-guided transluminal biliary drainage. Endoscopy.

[39] Bories E, Pesenti C, Caillol F, Lopes C, Giovannini M (2007). Transgastric endoscopic ultrasonography-guided biliary drainage: results of a pilot study. Endoscopy.

[40] Park do H, Song TJ, Eum J, Moon SH, Lee SS, Seo DW (2010). EUS-guided hepaticogastrostomy with a fully covered metal stent as the biliary diversion technique for an occluded biliary metal stent after a failed ERCP (with videos). Gastrointest Endosc.

[41] Mallery S, Matlock J, Freeman ML (2004). EUS-guided rendezvous drainage of obstructed biliary and pancreatic ducts: report of 6 cases. Gastrointest Endosc.

[42] Kahaleh M, Wang P, Shami VM, Tokar J, Yeaton P (2005). EUS-guided transhepatic cholangiography: report of 6 cases. Gastrointest Endosc.

[43] Lai R, Freeman ML (2005). Endoscopic ultrasound-guided bile duct access for rendezvous ERCP drainage in the setting of intradiverticular papilla. Endoscopy.

[44] Kahaleh M, Hernandez AJ, Tokar J, Adams RB, Shami VM, Yeaton P (2006). Interventional EUS-guided cholangiography: evaluation of a technique in evolution. Gastrointest Endosc.

[45] Maranki J, Hernandez AJ, Arslan B, Jaffan AA, Angle JF, Shami VM (2009). Interventional endoscopic ultrasound-guided cholangiography: long-term experience of an emerging alternative to percutaneous transhepatic cholangiography. Endoscopy.

[46] Kim YS, Gupta K, Mallery S, Li R, Kinney T, Freeman ML (2010). Endoscopic ultrasound rendezvous for bile duct access using a transduodenal approach: cumulative experience at a single center. A case series. Endoscopy.

[47] Shah JN, Marson F, Weilert F, Bhat YM, Nguyen-Tang T, Shaw RE (2012). Single-operator, single-session EUS-guided anterograde cholangiopancreatography in failed ERCP or inaccessible papilla. Gastrointest Endosc.

[48] Iwashita T, Lee JG, Shinoura S, Nakai Y, Park DH, Muthusamy VR (2012). Endoscopic ultrasound-guided rendezvous for biliary access after failed cannulation. Endoscopy.

[49] Dhir V, Bhandari S, Bapat M, Maydeo A (2012). Comparison of EUS-guided rendezvous and precut papillotomy techniques for biliary access (with videos). Gastrointest Endosc.

[50] Kawakubo K, Isayama H, Sasahira N, Nakai Y, Kogure H, Hamada T (2013). Clinical utility of an endoscopic ultrasound-guided rendezvous technique via various approach routes. Surg Endosc.

[51] Park do DH, Jeong SU, Lee BU, Lee SS, Seo DW, Lee SK (2013). Prospective evaluation of a treatment algorithm with enhanced guidewire manipulation protocol for EUS-guided biliary drainage after failed ERCP (with video). Gastrointest Endosc.

[52] Iwashita T, Lee JG (2012). Endoscopic ultrasonography-guided biliary drainage: rendezvous technique. Gastrointest Endosc Clin N Am.

[53] Nguyen-Tang T, Binmoeller KF, Sanchez-Yague A, Shah JN (2010). Endoscopic ultrasound (EUS)-guided transhepatic anterograde self-expandable metal stent (SEMS) placement across malignant biliary obstruction. Endoscopy.

[54] Artifon EL, Safatle-Ribeiro AV, Ferreira FC, Poli-de-Figueiredo L, Rasslan S, Carnevale F (2011). EUS-guided antegrade transhepatic placement of a self-expandable metal stent in hepatico-jejunal anastomosis. JOP.

[55] Park do H, Jang JW, Lee SS, Seo DW, Lee SK, Kim MH (2012). EUS-guided transhepatic antegrade balloon dilation for benign bilioenteric anastomotic strictures in a patient with hepaticojejunostomy. Gastrointest Endosc.

[56] Iwashita T, Yasuda I, Doi S, Uemura S, Mabuchi M, Okuno M (2013). Endoscopic ultrasound-guided antegrade treatments for biliary disorders in patients with surgically altered anatomy. Dig Dis Sci.

